# Advances and clinical challenges of mesenchymal stem cell therapy

**DOI:** 10.3389/fimmu.2024.1421854

**Published:** 2024-07-19

**Authors:** Ruiyan Mei, Zhuo Wan, Cheng Yang, Xiangjing Shen, Ronglin Wang, Haihua Zhang, Rui Yang, Junqiang Li, Yang Song, Haichuan Su

**Affiliations:** ^1^ Department of Oncology, Tangdu Hospital, Air Force Medical University, Xi’an, China; ^2^ Department of Hematology, Tangdu Hospital, Air Force Medical University, Xi’an, China

**Keywords:** mesenchymal stem cell, cell homing, drug delivery, nanoparticles, clinical use

## Abstract

In recent years, cell therapy has provided desirable properties for promising new drugs. Mesenchymal stem cells are promising candidates for developing genetic engineering and drug delivery strategies due to their inherent properties, including immune regulation, homing ability and tumor tropism. The therapeutic potential of mesenchymal stem cells is being investigated for cancer therapy, inflammatory and fibrotic diseases, among others. Mesenchymal stem cells are attractive cellular carriers for synthetic nanoparticles for drug delivery due to their inherent homing ability. In this review, we comprehensively discuss the various genetic and non-genetic strategies of mesenchymal stem cells and their derivatives in drug delivery, tumor therapy, immune regulation, tissue regeneration and other fields. In addition, we discuss the current limitations of stem cell therapy and the challenges in clinical translation, aiming to identify important development areas and potential future directions.

## 1 Introduction

Mesenchymal stem cells (MSCs) are multipotent cells with the potential to differentiate into mesenchymal cell lines. Compared with embryonic stem cells and induced pluripotent stem cells, MSCs have no ethical issues and no risk of teratoma formation, and have gained more and more clinical appeal in recent years (1). Drug-loaded and genetically modified mesenchymal stem cells and their derivatives have been shown to deliver drugs and therapeutic cytokines to the site of injury (2, 3). MSCs have been reported to be used in neural differentiation, inflammation reduction and various tumor models, such as melanoma (4), colon cancer (5), pancreatic cancer (6), breast cancer (7)and hepatocellular carcinoma (8).

Chimeric antigen receptors (CAR) are widely used on T cells, natural killer cells, dendritic cells and macrophages (9). Despite their efficacy, CAR-related therapies face challenges such as poor persistence of CAR-carrying cells, cytokine release syndrome, and cell dysfunction. Recent studies have shown that MSCs can enhance the function of CAR-based therapies and help overcome these drawbacks, improving the overall effectiveness of cell therapy. Sirpilla et al. used an E-cadherin-targeted chimeric antigen receptor to construct CAR-MSCs to enhance immunosuppressive potency at sites of inflammation while maintaining their stem cell phenotype and safety in an animal model (10, 11). Aliperta et al. used MSCs as autonomous cellular machines for the continuous production of the most humanized anti-CD33-anti-CD3 bsAb, enabling it to redirect human T cells against CD33-expressing leukemic cells (12). This study suggests that MSC-modified CAR has greater lethality while regulating the proliferation of other immune cells such as T cells. CAR-MSCs are a therapeutic technology that can be widely used to enhance immunosuppression.

In recent years, the research on biomimetic membranes has gradually increased. The cell membrane of stem cells and stem cell-derived extracellular vesicles are considered to be effective natural carriers. Since the cell membrane of stem cells and the extracellular vesicles derived from stem cells preserve membrane proteins related to intercellular communication and immune regulation, the prepared biomimetic materials not only have their unique biological properties, but also preserve the physical and chemical properties of the source cells (13). The drug delivery system based on the membrane and extracellular vesicles of MSCs not only enhances the biocompatibility, but also maximizes the therapeutic effect of biomaterials by simulating the targeting ability of MSCs (14). These characteristics have gradually attracted the attention of researchers and have been applied to the treatment of diseases. In this review, we provide a comprehensive overview of the research progress of MSCs and their derivatives in various diseases. In addition, we analyzed and discussed the challenges of MSCs and their derivatives in clinical applications.

## 2 The origin and mechanism of MSCs

### 2.1 Culture and differentiation

**Figure 1:**
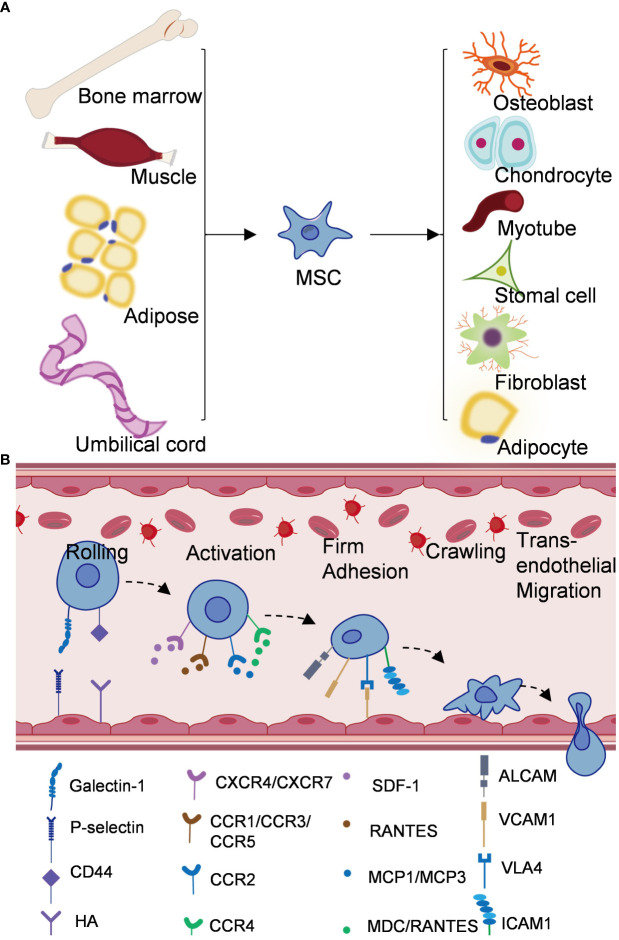
Origin and mechanism of mesenchymal stem cells. **(A)** Mesenchymal stem cells come from bone marrow, adipose tissue, umbilical cord, muscle and other tissues, and can differentiate into different cell types. **(B)** Schematic diagram of homing mechanism of mesenchymal stem cells.

In the 1970s, Friedenstein et al. first identified mesenchymal stem cells as spindle-shaped, adherent non-hematopoietic stem cells in bone marrow (15, 16). MSCs have been obtained from bone marrow, cord blood, placenta, heart, adipose tissue, synovial tissue and other tissues (17, 18) (**Figure 1**). Currently, the markers of MSCs lack absolute specificity and there are no clear phenotypic markers. According to the International Society for Cell & Gene Therapy (ISCT), when mesenchymal stem cells were detected by flow cytometry, the expression rates of CD105, CD90, CD73 were found to be over 95%, and the expression rate of CD45, CD34 was less than 5%, while meeting the standard culture conditions for plastic adhesion and demonstrating differentiation potential into osteoblasts, adipocytes, and chondroblasts, the obtained samples can be considered as mesenchymal stem cells (19–21). Sakaguchi et al. isolated mesenchymal stem cells from different tissues and compared their colony forming capacity and differentiation under defined conditions, and found that bone marrow, synovial and periosteum-derived mesenchymal stem cells had the highest alizarin red positive rate in osteogenesis, and synovial mesenchymal stem cells had the greatest ability for chondrogenesis (22). These results showed that MSCs derived from different tissues differ in their differentiation capacity even when cultured under the same culture conditions. The number of bone marrow derived-MSCs decreases dramatically with age, and fetal MSCs have a higher proliferative capacity (23). Baxter and Liu et al. found that even minimal expansion induced a rapid aging of MSCs and that telomerase activity in MSCs is required not only for self-replication but also for differentiation (24, 25). MSCs often lack major histocompatibility complex (MHC)‐II and co-stimulatory molecules (17, 26, 27). Numerous studies have demonstrated that MSCs can avoid allogeneic rejection in humans and different animal models, which opens up broader prospects for the clinical application of MSCs, rather than only using autologous cell sources (28). MSCs may also have the ability to regulate the differentiation, maturation and function of dendritic cells. some studies have shown that MSCs can induce differentiation of mDC into DCregs, along with reduce expreesion of MHCII, CD11C, CD80, CD86 and CD40 (26, 29, 30). a et al. showed that the expression of CD83 in mature DCs treated with MSCs was significantly reduced, suggesting that DCs were tilted toward the immature state, and MSCs could inhibit monocyte differentiation into DCs (26).

The differentiation of MSCs is a two-step process, the process from MSCs to progenitor cells with lineage specificity and the process from progenitor cells to specific cell types (18). Studies in recent decades have shown that many signaling pathways are involved in the regulation of mesenchymal stem cell lineage differentiation, including transforming growth factor-beta (TGF-β)/bone morphogenic protein (BMP) signaling, fibroblast growth factors (FGFs), Notch signaling and Hedgehogs signaling, etc (16, 31). TGF/BMPs signaling has been generally recognized to play a role in both adipogenesis and osteogenesis differentiation of MSCs. BMP4 can promote the adipogenic differentiation of MSCs, while high dose of BMP2 can promote the osteogenic and chondrogenic differentiation of C3H10T1/2 (32, 33). Several studies have shown that the FGF receptor signaling cascade involves ERK1/2, p38MAPK, SAPK/JNK, PCK and PI3K pathways, all of which have been shown to play important roles in regulating osteogenic and adipogenic differentiation of MSCs (34, 35). The activation of Wnt signaling promotes osteogenic differentiation and inhibits adipogenic differentiation of MSCs. Bennett et al. demonstrated that activation of Wnt signaling by overexpression of Wnt10b increased the thickness of trabecular bone, lack of Wnt10b resulted in decreased bone density, they also suggested that the reduction of Wnt10b was associated with an increase in aging-associated adipocytes (36). Colter et al. found that culturing MSCs at low densities (1.5 or 3.0 cells/cm^2^) led to more rapid proliferation, the MSCs entered a phase of rapid exponential growth after approximately 5 days and maintained their multipotentiality (37, 38). Besides, Gregory et al. found that MSCs secreted large amounts of Dickkopf-1, an inhibitor of the Wnt signaling pathway, when it left the lag phase. Subsequently, Gregory added recombinant Dickkopf-1 after the end of the lag period to increase cell proliferation, suggesting that high levels of Dickkopf-1 allowed cells to re-enter the cell cycle by inhibiting the Wnt/β-catenin signaling pathway (39). Recently, it has been shown that blocking Notch signaling pathway can promote autophagy-mediated adipogenic differentiation of MSCs via PTEN-PI3K/AKT/mTOR pathway (40). Notch signaling can also interact with BMP2 signaling to promote osteogenic differentiation (41). Hedgehog signaling can promote osteogenic and adipogenic differentiation, and the mutual interference between Hedgehog pathway and BMP signal can regulate Smad signaling to promote osteogenic differentiation (42). These signaling pathways can be activated simultaneously by stimuli from specific microenvironments. In addition, a variety of microRNAs, transcription factors, biological factors, chemical factors and physical factors play important roles in the differentiation of MSCs (18). These factors eventually converge in a tightly controlled cascade of events that affect the balance between adipogenic and osteogenic differentiation of MSCs.

### 2.2 Homing

A key advantage of MSC-based therapy is its ability to preferentially homing to damaged tissues. Homing of MSCs is thought to be important in tissue regeneration, which reflects the ability of stem and progenitor cells to recruit and homing to damaged tissues in need of repair (37). Different from the process of leukocyte migration to inflammatory sites, MSCs homing is divided into non-systemic homing and systemic homing (43). Unsystematic homing refers to the local transplantation of mesenchymal stem cells to the damaged site, while systematic homing refers to the migration of MSCs to the target tissue through vascular endothelial cells under the guidance of homing promoting factors released by the damaged tissue (44). This process is divided into five steps (**Figure 1**) (1): Rolling, Ruster et al. found that MSCs bind to endothelial cells in a P-selectin dependent manner, however, MSCs do not express P-selectin ligands, indicating that other ligands interact with P-selectin on the surface of MSCs (45). Studies have shown that glycoproteins and galectin-1 expressed on bone marrow MSCs have been identified as alternative P-selectin ligands (27). CD44 receptor is also known as homing receptor, and studies have shown that hyaluronic acid is a potential binding site of CD44 receptor-mediated MSCs homing (46) (2). Activation, stromal cell-derived factor-1 (SDF-1), a small chemokine in the C-X-C motif chemokine family, plays a key role in MSCs transportation and homing (47). SDF-1 expression in injured blood vessels and tissues upregulates SDF-1 binding to CXCR4 expressed by MSCs and induces MSCs to mobilize and homing along the SDF-1 concentration gradient to the damaged tissues and exert their effects (48). In addition, CXCR7 was also identified as a receptor for SDF-1 and involved in MSCs homing (43) (3). Firm adhesion: after entering the peripheral blood circulation, mesenchymal stem cells continuously roll with vascular endothelial cells (49). VLA4/VCAM1 plays a key role in the firm adhesion between MSCs and endothelial cells (45). VLA4 expressed by MSCs is activated by chemokines such as SDF-1 and binds to VCAM1 on endothelial cells to activate the cell adhesion signaling pathway and promote the adhesion of MSCs to endothelial cells (49, 50) (4). Crawling, CCR2/FROUNT/PI3K signaling pathway promotes the formation of actin filaments and pseudopodia, thereby mediating cytoskeletal reorganization and promoting MSCs to crawl and migrate in the inner wall of blood vessels (51, 52) (5). Transendothelial migration. MSCs secrete matrix metalloproteinases (e.g., MMP9, MT1-MMP) and proteolytic enzymes (e.g., uPA) to disrupt the barrier of the endothelial basement membrane and pericyte sheath to complete transepithelial migration (53–55). Improving the homing efficiency of MSCs is one of the challenges in MSCs therapy. At present, there are targeted drug delivery, genetic engineering of stem cells, magnetic guidance and other technologies, aiming to improve the systematic or non-systematic homing ability of MSCs through various technologies (56, 57).

## 3 The role of MSCs as drug delivery vehicles in diseases of different systems

**Figure 2:**
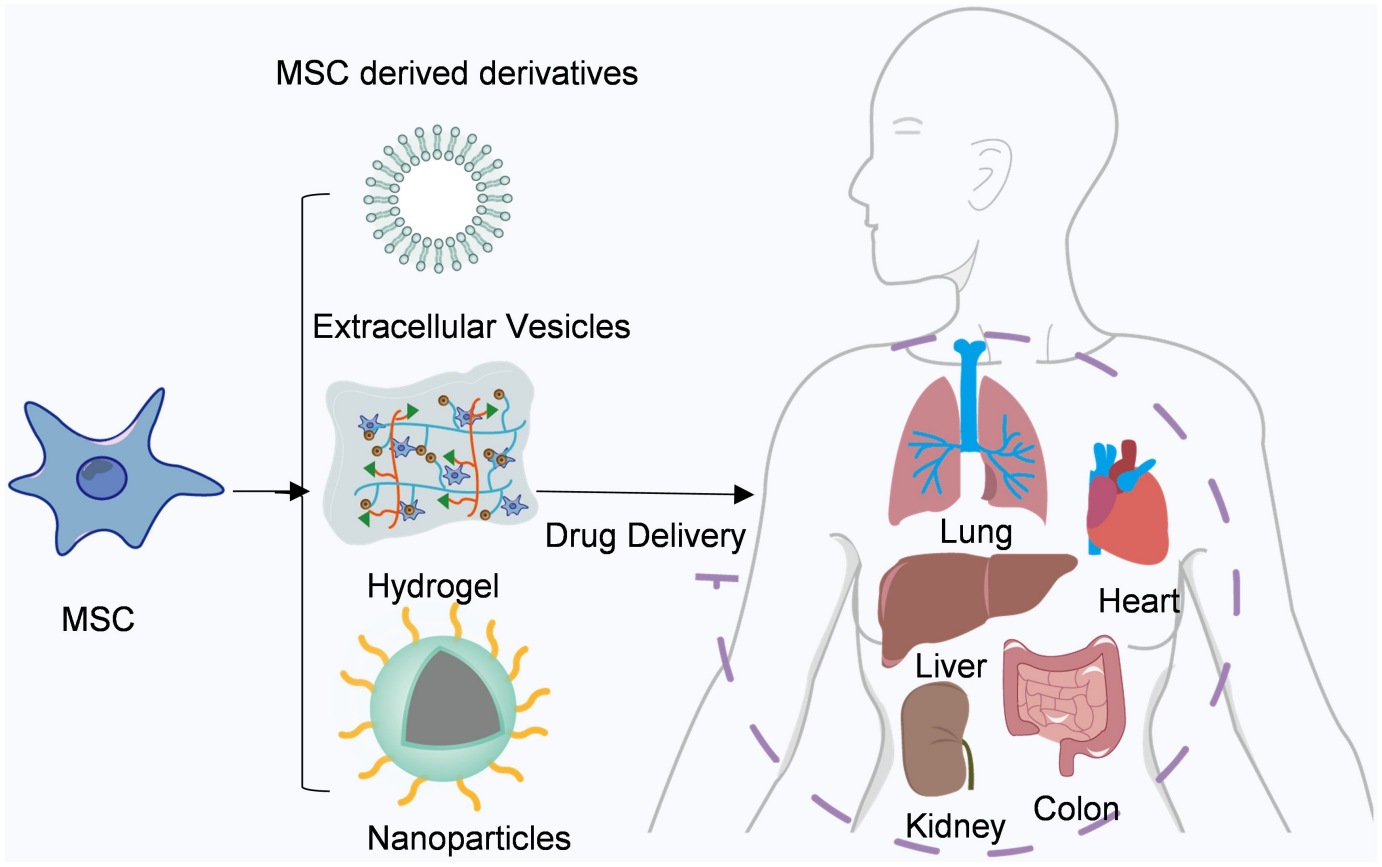
Mesenchymal stem cells and their derivatives are used for drug delivery. Mesenchymal stem cells and their derivatives can deliver a variety of drugs to different tissues throughout the body, such as the heart, liver, and lung.

In recent years, mesenchymal stem cell therapy has emerged as a prominent field in the domains of anti-tumor treatment and tissue regeneration. Owing to their inherent capacity for autonomous differentiation, facile isolation and propagation, as well as attenuation of immune effector responses, numerous ongoing clinical trials are underway (58). Their utilization has witnessed a steady rise in graft-versus-host disease (GVHD) and autoimmune disorders such as lupus and Crohn’s disease (59, 60). Furthermore, the clinical therapeutic potential of MSCs has been extended to encompass myocardial infarction, stroke, multiple sclerosis, liver cirrhosis, diabetes, lung injury, and cancer (61–64) (**Figure 2**).

### 3.1 Cancer

Cancer is a major public health problem worldwide, threatening human health (65). Addressing tumor treatment has emerged as an urgent medical imperative. Numerous investigations have consistently demonstrated the involvement of mesenchymal stem cells in cancer initiation, development, progression, and metastasis (28, 49, 57). Furthermore, research has substantiated the immunomodulatory properties of mesenchymal stem cells along with their capacity to selectively migrate towards inflammatory and neoplastic sites, rendering them promising vehicles for targeted delivery of anti-tumor therapeutics (50, 66).

Allogeneic bone marrow or hematopoietic stem cell transplantation is a crucial therapeutic approach for leukemia, multiple myeloma, and lymphoma (67). Following hematopoietic stem cell transplantation, GVHD frequently occurs in patients (68, 69). MSCs have been reported to possess immunosuppressive properties that not only alleviate GVHD but also enhance hematopoietic reconstitution post-transplantation (70). Although some studies have suggested the pro-tumor function of MSCs, it is generally accepted that MSCs can impede tumor growth through various mechanisms such as disrupting tumor cell cycle and inducing apoptosis (71). Secchiero and Ho et al. demonstrated that MSCs can suppress tumor angiogenesis by downregulating the platelet-derived growth factor/platelet-derived growth factor receptor (PDGF/PDGFR) axis in glioma cells (72). By co-culturating umbilical cord derived mesenchymal stem cells with glioblastoma cancer stem cells, Bajetto et al. found that direct interaction (cell-to-cell contact) would cause inhibitory reactions, and the proliferation of both types of cells would be slowed down, while indirect interaction (via the release of soluble factors) would cause irritant reactions (73). Meanwhile, Sarmadi et al. showed that MSCs hinder the progression of lymphoid hematopoietic tumor cells by arresting them at the G0/G1 phase through intercellular contact (74). Despite reports on the regulatory role of MSCs in Wnt signaling within tumors, there remains controversy regarding whether this regulation suppresses or enhances tumor development. On one hand, studies have indicated that β-catenin—an essential signaling molecule in the Wnt pathway—enhances telomerase reverse transcriptase (TERT) expression and prevents telomere loss in cancer stem cells (75). Conversely, MSCs secrete Dickkopf-1 to modulate Wnt signaling and attenuate leukemia tumor cell proliferation (76).

As carriers of anti-tumor drugs, MSCs can be genetically engineered to express or secrete a variety of therapeutic agents that inhibit cancer growth and progression. These agents encompass therapeutic proteins, suicide genes, and oncolytic viruses (4). Cytokines and growth factors have been identified as crucial regulatory factors in tumor development, therefore, therapeutic proteins based on cytokines and growth factors that impede tumor growth or act as inhibitors of pro-tumor factors have emerged as potential anti-tumor drugs (77). MSCs are considered an ideal vehicle for delivering these therapeutic proteins. Wong et al. demonstrated the anti-proliferative and apoptotic effects of gene-edited MSCs capable of producing IFN-β on tumor cells (78). Several studies have shown that the expression of the pro-apoptotic protein tumor necrosis factor-related apoptosis-inducing ligand (TRAIL) in MSCs can induce the apoptosis of tumor cells in various tumors, including breast cancer, lung cancer, colorectal cancer, and cervical cancer (79–81). IL-12 is regarded as an optimal anti-tumor factor due to its ability to stimulate T cell and NK cell activation, Gao and Seo et al.’s research reported that IL-12-expressing MSCs can suppress the occurrence and progression of renal cell carcinoma and cervical cancer in mice (66, 82).

In addition, MSCs are often engineered to secrete suicide genes, converting non-toxic reagents into toxic anti-tumor drugs (83). By employing gene-directed enzyme prodrug therapy, the systemic toxicity of 5-FU can be circumvented by harnessing the enzymatic activity of bacterial and/or yeast cytosine deaminase (CD) to convert the less toxic substrate 5-fluorocytosine (5-FC) into 5-FU (84). Lucia et al. demonstrated the targeted migratory capacity of CD-AT-MSCs towards tumor cells and their ability to inhibit tumor growth in tissues through the generation of adipose tissue-derived mesenchymal stem cells (AT-MSCs) expressing a fusion gene consisting of yeast cytosine deaminase and uracil phosphate riboside transferase (CD-AT-MSCs) (83). By targeting tumor cell surface proteins, oncolytic viruses (OVs) can bind to tumor cells and lead to oncolytic effect (85). The administration of OVs via MSCs has been proven effective in treating lung metastasis in glioblastoma, hepatocellular carcinoma, and breast cancer (86–89). Zhang et al. discovered that oncolytic herpes simplex virus-1 modulates the tumor microenvironment by reducing macrophage percentages while increasing tumor-infiltrating lymphocyte numbers (90). Furthermore, combining oncolytic viruses with immune checkpoint modulators significantly prolongs survival in mice bearing tumors (91).

### 3.2 Immunomodulation

To date, mesenchymal stem cells have demonstrated immunomodulatory effects through direct intercellular contact and paracrine secretion of soluble factors, including modulation of lymphocyte proliferation, inhibition of dendritic cell activation, regulation of B cell proliferation and function, induction of regulatory T cell proliferation, and suppression of natural killer cell activation (28, 92–94). *In vitro*, MSCs have been shown to inhibit naive and memory T cells from communicating with antigen-presenting cells by upregulating intercellular adhesion molecule-1 (ICAM-1) and vascular cell adhesion molecule-1 (VCAM-1), while ICAM-1 is key to T cell activation and leukocyte recruitment to inflammatory sites (77). In addition, mesenchymal stem cells expressing Toll-like receptor 3 and Toll-like receptor 4 have been shown to restore effective T cell responses in the presence of infection (95). Francesa et al. reported that MSCs increase the survival of quiescent B cells through a contact-dependent mechanism and promote B cell differentiation independent of T cells (96). Furthermore, MSCs supported CD19(+) B cell proliferation through a cell contact dependent mechanism, promote AKT phosphorylation in B cells by increasing vascular endothelial growth factor (VEGF) production, and inhibit Caspase 3-mediated apoptosis (97). Studies on the co-culture of MSCs with different types of NK cell lines (KHYG-1 and NK-92) showed that MSCs inhibited the cytotoxic activity of KHYG-1 in both direct and indirect co-culture, and inhibited the cytotoxic activity of NK-92 in indirect co-culture, but direct contact had no effect on the cytotoxic activity of NK-92 (98). Further studies indicated that MSC-mediated cytotoxic activity depended on the differential crosstalk between the two types of cells (98). In the innate immune system, MSCs interact with NK cells by inhibiting IL-2-induced proliferation of NK cells and inducing cytotoxic activity or cytokine production through secretion of IDO (Indoleamine-2,3-Dioxygenase) and PGE-2 (Prostaglandin E2) (92, 99). Additionally, IL-6 secreted by MSCs can prevent monocytes from differentiating into IL-10-producing phenotypes, while MSC-derived PGE2 enables inhibition of monocyte differentiation into mature dendritic cells (DCs) (100, 101). Furthermore, miR-21–5p, which enriched in extracellular vesicles derived from MSCs, has been shown to affect the maturation and function of DCs (102). Preconditioning MSCs with hypoxia and immunomodulatory factors has also been demonstrated to increase their potential for survival and efficacy through increased paracrine effects and antioxidant activity as well as observed angiogenic factor secretion in acute kidney injury and bleomycin-induced pulmonary fibrosis (103–105). Moreover, membrane particles (MPs) derived from IFN-γ stimulated MSCs have been found to increase mRNA expression of Programmed Cell Death Ligand 1 (PD-L1) in monocytes as well as the percentage of anti-inflammatory PDL-1 and CD90-positive monocytes suggesting potential use for MP-based cell-free therapy for immune diseases (106). Finally, transforming MSCs to express specific immunomodulators such as IFN-γ or interleukin can enhance their inherent ability to temporarily escape the immune response while increasing their pluripotency leading to tumor growth reduction through polarization towards pro-inflammatory M1 phenotype (107, 108). It has been shown that mesenchymal stem cells have intrinsic immunosuppressive abilities that can reduce inflammation and immune responses. Because mesenchymal stem cell-derived EVs have similar biological functions as MSCs and are more stable and less immunogenic, MSCs-EVs can be an excellent alternative to MSCs (109). Fan et al. used MSC-EVs to treat diabetic mice, and the results showed that MSC-EVs treatment reduced M1 macrophage phenotype markers and increased M2 macrophage phenotype markers, and MSC-EVs alleviated neurovascular dysfunction in diabetic peripheral neuropathy mice by inhibiting pro-inflammatory genes (110). Mathew et al. reported that injecting MSCs-EVs into the vitreous body can significantly improve retinal functional recovery and reduce neuroinflammation and apoptosis (111).

### 3.3 Fibrotic diseases

One of the reasons for organ fibrosis is the plasticity of fibroblasts. Research has demonstrated that adipocytes and adipose-like cells transform into collagen-secreting myofibroblasts during the development of fibrosis in the lung, liver, and skin (27). Myofibroblasts are the primary cells involved in fibrosis, and their activation can be triggered by various mechanisms, including paracrine signals from macrophages and lymphocytes or autocrine signals derived from myofibroblasts themselves (112). Schermulty et al. genetically engineered myofibroblasts formed during the development of fibrosis to regenerate into adipose-like cells and fat cells in the lung (113). Given the multiline differentiation potential of MSCS, studying the fate of MSC-derived myofibroblasts may provide valuable hints for future anti-fibrotic therapies (114). Exogenous mesenchymal stem cells possess anti-fibrotic properties (115). TGF-β is considered as a key regulator of fibrosis, exerting its effects through two signaling pathways: the classical pathway dependent on Smad2/3 and the non-classical pathway independent of Smad signaling (116). In a mouse model of CCL4-induced liver cirrhosis, infusion of bone marrow-derived MSCs can reduce liver fibrosis area by decreasing TGF-β levels while enhancing BMP7 levels (117). Jiang et al. demonstrated that bone marrow-derived mesenchymal stem cells inhibit thioacetamide-induced liver fibrosis progression by suppressing the TGF-β/Smad signaling pathway or directly inhibiting hepatic stellate cell proliferation through upregulation of Notch1 expression and downregulation of PI3K/AKT or Wnt/β-catenin pathways, thereby reducing liver fibrosis (26). Tonsil-derived mesenchymal stem cells exert their anti-fibrotic effects in liver fibrosis by downregulating the expression of collagen I and TGF-β through an autophagy-dependent mechanism (118). Pulmonary fibrosis is characterized by excessive deposition of extracellular matrix (ECM), destruction of lung parenchyma and epithelial barrier, as well as progressive proliferation of fibroblasts and myofibroblasts (116). Adipose tissue-derived MSCs mitigate bleomycin-induced pulmonary fibrosis in rats by modulating IL-17-mediated immune responses induced by ECM (119). Bone marrow-derived MSCs possess anti-fibrotic properties attributed to their ability to release IL-1 receptor antagonists, leading to a reduction in the expression and activity of IL-1 and TNF-α (120). Bone marrow-derived MSCs alleviate renal interstitial fibrosis in a model of unilateral ureteral obstruction by inhibiting capillary dysfunction around renal tubules, promoting parenchymal cell proliferation, and suppressing myofibroblast activation and differentiation (121). Yong et al.’s study revealed that hepatocyte growth factor (HGF) present in conditioned medium from adipose-derived MSCs hinders cardiac fibroblast differentiation into myofibroblasts via inhibition of angiotensin II type 1 receptor signaling pathway while upregulating inhibitory Smad7 (28, 122). TGF-β binding receptor activates sphingosine kinase 1/1-sphingosine phosphate/mammalian target of rapamycin (SPHK1/S1P/mTOR) pathway and accelerates the production of pro-fibrosis molecules, ultimately leading to the occurrence of intestinal fibrosis (123). Kang and Wang et al. reported that MSCs release miR-148b-5p, which suppresses the expression of 15-lox-1 in macrophages, thereby attenuating inflammatory bowel disease by inhibiting ERK phosphorylation in neutrophils (124, 125). A study demonstrated that both intravenous infusion and injection of MSCs after anal sphincter injury in rats significantly reduced fibrosis and scar tissue compared to the PBS treatment group (126). Intestinal fibrosis is a common complication of anal fistula. Injecting autologous adipose tissue-derived mesenchymal stem cells completely cured 57% of fistula patients and reduced secretion in some remaining patients (127). Autologous and allogeneic adipose tissue-derived mesenchymal stem cells have shown favorable results with high safety in long-term clinical trials for fistula treatment (128, 129). In various fibrotic diseases, MSC-mediated anti-fibrotic activity appears to involve the TGF-β/Wnt/Smad signaling pathway as a common mechanism. Enhancing the homing ability of mesenchymal stem cells is crucial for improving their therapeutic efficacy. Liao et al. utilized microbial orthogonal reaction to modify liver sinusoidal endothelial cell (LESC)-targeting peptide RLTRKRGLK on adipose-derived mesenchymal stem cells, and demonstrated that the modified MSCs had higher liver-targeting delivery efficiency, significantly enhancing liver regeneration and anti-inflammatory effects (130). Vittorio et al. injected MSCs loaded with carbon nanotubes into the portal vein of rats to investigate the effect of magnetic force exerted by carbon nanotubes on MSCs homing, and the results showed that carbon nanotubes could guide MSCs to migrate *in vivo* and *in vitro*, increasing their transplantation and homing in liver tissue (131, 132).

## 4 MSCs and their derivatives are used as drug delivery vehicles

Drug-loaded and genetically modified mesenchymal stem cells can deliver drugs and therapeutic cytokines to sites of injury or inflammation, thus having applications in regenerative and antitumor therapies. From cell-derived peptide modification to cell-based drug delivery systems, genetic engineering and biomimetics provide a new strategy of nanoparticles, which are attractive candidates for drug therapy. Here, we summarize some of the studies on the use of MSCs and their derivatives in the treatment of diseases (**Table 1**).

**Table 1:** Application of MSCs and its derivatives in the treatment of diseases.

Form of drug	Applications	Interventions	Reference
Engineered MSC	Breast cancer, lung cancer, colorectal cancer, and cervical cancer	TRAIL-expressing MSCs	(79–81)
	Renal cell carcinoma and cervical cancer	IL-12-expressing MSCs	(66, 82)
	Disseminated neuroblastoma	MSCs loaded drugs	(83)
	Liver injury	Targeting peptide-expressing MSCs	(130)
	Liver fibrosis	MSCs loaded carbon nanotubes	(131, 132)
MSC-derived extracellular vesicles	Breast cancer, osteoarthritis	MSCs-derived EVs loaded miRNA	(133, 134)
	Pancreatic cancer,	MSCs-derived EVs loaded siRNA	(135)
	Cerebral ischemia-reperfusion injury	MSCs-derived EVs loaded mRNA	(136)
	Colorectal cancer, thyroid cancer, inflammatory diseases	MSCs-derived EVs loaded drugs	(135, 137, 138)
MSC with hydrogels	Bone defect	Integrin-specific hydrogels	(139)
	Hindlimb Ischemia	chitosan hydrogel	(140)
	Spinal cord injury	3D-exohydrogel hybrid microneedle array patch	(141)
	Diabetic Wound	Pluronic F127 Hydrogel	(142)
	Peri-implantitis	MSC-encapsulated Adhesive hydrogels	(143)
	Calvarial defects	MSC-encapsulated HPCH + poly (ϵ-caprolactone)/nano-hydroxyapatite (PCL/nHA)	(144)
MSC cell membrane-coated nanoparticles	Orthotopic glioma	PTX-PLGA-loaded MSCs	(145)
	Liver injury, prostate tumor	SPIO@AuNPs- loaded MSCs	(146, 147)
	Heart Repair	NA@MEV-loaded MitoN	(148)
	Immune diseases	IFN-γ-expressing membrane particles	(106–108)

### 4.1 MSC-derived extracellular vesicles

Extracellular vesicle therapy based on mesenchymal stem cells has been proven to be biosafe, highly stable and low immunogenic. Studies have shown that bone marrow mesenchymal stem cells can regulate the fate of tumor cells in a paracrine manner (149). MSC-derived extracellular vesicles (EVs) have a strong ability to migrate to tumor sites, so MSC-derived EVs are the main contributors to paracrine. MSC-derived EVs are widely used as safe and versatile drug delivery platforms.

Naseri et al. loaded anti-miR-142–3p LNA into EVs derived from mouse BMMSCs by electroporation (133). Small interfering RNAs (siRNAs) and drugs can also be loaded into extracellular vesicles by electroporation. Zhou et al. reported an exosome-based dual delivery biological system, IEXO-OXA. By loading galectin-9 siRNA into exosomes and surface modification of exosomes with oxaliplatin prodrug as an immunogenic death trigger, therapeutic exosomes can improve tumor targeting and increase drug accumulation at tumor sites (150). Bagheri et al. loaded doxorubicin into exosomes derived from mouse BMMSCs by electroporation and showed that tumor volume was reduced and survival was prolonged in tumor-bearing mice (135). Bliss et al. used ultrasonically treated hADMSC-derived EVs loaded with tyrosine kinase inhibitors and found a significant decrease in iodine affinity of iodine-resistant thyroid cancer cells (137). Currently, MSC-derived EVs have been used in clinical trials, and 17 patients recovered after treatment with mesenchymal stem cells derived exosomes (MSC-EXOs) in 24 patients with COVID-19 disease (149). A clinical trial (NCT01547091) conducted at the PLA Air Force Hospital showed that MSC combined with low-dose antirheumatic drugs had a long-term beneficial effect in patients with rheumatoid arthritis, which was better than that in patients treated with antirheumatic drugs alone (138). Wang et al. co-cultured TGF-β1-MSC- EXOs with rat chondrocytes and found that the expression of specific protein 1 (SP1) in chondrocytes was decreased and the proliferation activity of chondrocytes was enhanced (134).

MSC-derived EVs also have therapeutic limitations. The most suitable MSC source may need to be screened for the production of EVs, and the long-term safety and therapeutic efficacy of MSC-derived EVs need to be further verified. In addition, MSC-derived EVs require a larger dose but have a lower yield. Future advances in nanotechnology still require attention and issues such as breakthrough yield and stability.

### 4.2 MSC with hydrogels

Hydrogels are hydrophilic, three-dimensional and cross-linked polymer networks that are highly adjustable materials (151). Currently, hydrogel systems have been designed to promote MSCs proliferation and maintain stem cell dryness during *in vitro* expansion, or to improve MSCs survival, retention, and implantation *in vivo* (152). The researchers influenced the fate and secretion of MSCs by altering the properties of the hydrogel, such as stiffness, viscoelasticity, porosity, and degradability (139, 153, 154).

Since integrin αβ heterodimers of the cellular ECM adhesion receptor family regulate cell anchoring, migration, and survival, Amy et al. prolonged the survival, implantation, and secretion and repair activities of MSC in tissues by presenting a specific peptide targeting α2β1 integrin in the hydrogel (139). Zhang et al. loaded MSC- EXOs into chitosan hydrogel, which not only improved the stability of proteins and miRNAs in MSC-EXOs, but also prolonged their retention time *in vivo* (140). Traditional two-dimensional culture inevitably leads to the loss of stem cells, which limits the therapeutic efficacy of MSC-EXOs. Han et al. proposed a controlled 3D-exohydrogel hybrid microneedle array patch to achieve constant *in situ* release of exosomes. The results showed that 3D-cultured MSCs could maintain their stemness. The obtained exosomes can effectively reduce the inflammation and glial scar formation induced by spinal cord injury treatment, and realize the repair of spinal cord injury *in situ* (141). Human umbilical cord MSC-derived exosomes encapsulated in thermosensitive PF-127 hydrogel can significantly accelerate wound healing rate, promote granulation tissue regeneration, up-regulate the expression of VEGF and TGFβ-1, and increase wound healing growth factor (142). Currently, cell-containing hydrogels have been widely used in tissue engineering and regenerative medicine, the moist and highly dynamic environment of the mouth makes the application of hydrogels in the local treatment of oral diseases a challenge (151, 155). Based on this, Mohammad et al. designed a hydrogel material with adjustable mechanical properties and biodegradability that can effectively provide patient-derived dental derived MSCs to promote the regeneration of craniofacial bone tissue in rats (143). One disadvantage of biodegradable hydrogels is that they are too weak to hold their shape, so designing hydrogels with both high cellular compatibility and good mechanical properties will enhance their application value. Ji et al. combined 3D printed customized poly (ϵ-caprolactone) (PCL) and natural hydroxypropyl chitin hydrogel (HPCH) to produce hydrogel not only has good cell compatibility, but also improves mechanical properties (144). Considering that implanted biomaterials may induce inflammation and local tissue damage by activating macrophages, Ji et al. also examined the interaction between the hydrogel and the immune response, showing that the hydrogel can regulate the transformation of macrophages to M2, possibly enhancing bone healing (144).

### 4.3 MSC cell membrane-coated nanoparticles

Over the past few decades, the application of biomedical nanotechnology in the treatment of diseases has continued to evolve. Inspired by the synthesis of liposomes, nanoparticles were prepared by extrusion through a porous polycarbonate membrane after separation of cell membranes by hypotonic, repeated freezing and thawing, or ultrasonic fragmentation (156). Microfluidic and electroporation techniques have also been applied to facilitate the synthesis of cell membrane-coated magnetic nanoparticles (157). Due to the asymmetric charge of the cell membrane, negatively charged nanoparticles are better able to form a membrane cover (158, 159). Drug-loaded nanoparticles can be taken up by cells and released continuously in cells (160). Polylactic acid-glycolic acid (PLGA) has been approved by the US Food and Drug Administration due to its high biodegradability and biocompatibility (13).

Pacioni et al. reported that chemotherapy drug Paclitaxel (PTX)-PLGA-loaded MSCs had a tropism to orthotopic glioma, and the release of the drug causes specific cytotoxic damage to tumor cells and had a sustained release effect (145). In addition, MSCs have been reported to uptake diagnostic nanoparticles to specific targets. Superparamagnetic iron oxide (SPIO) can be used to track labeled cells in cell therapy (161). Gold nanoparticles (AuNPs) are widely used as photothermal agents for tumor photothermal therapy (162, 163). SPIO@AuNPs can be used as a good diagnostic agent for magnetic resonance imaging (146). Adipose tissue-derived MSCs carry more SPIO@AuNPs at sites of liver injury or tumor and effectively kill surrounding cells *in vivo* (146, 147). Wang et al. used MSCs as lung-targeting vectors loaded with nanoparticles containing doxorubicin to verify the efficient targeting of MSCs in three animal models, namely mice, rabbits and monkeys, and the MSC/Nanoparticle (NP) delivery system effectively inhibited the development of lung cancer with a lower dose of anticancer drugs (3). Zhang et al. prepared macrophage bionic and nitric oxide (NO)-driven MSC-EV nanomodels NA@MEV that colonized damaged blood vessels, and a reactive oxygen species (ROS) scavenger called MitoN was used to mimic nicotinamide adenine dinucleotide (NADH) to attenuate ROS-induced DNA damage and DNA damage response (DDR), reinitiated myocardial proliferation, and triggered immunomodulation and proangiogenesis (148).

Although a large body of scientific literature on membrane-coated nanoparticles has demonstrated convincing therapeutic effects on different diseases, membrane coating technology still faces several challenges (164). The coated and uncoated nanoparticles are difficult to separate due to the limitation of the preparation method. The physiological mechanisms at the cellular level require a strong understanding, including membrane properties, drug forms and membrane-coated nanoparticle-environment interactions, drug release processes, and material loss during manufacturing.

## 5 Challenges in the clinical translation of MSCs

Mesenchymal stem cells have been widely studied as a kind of cell therapy, which shows good application prospects in tissue repair and regeneration, anti-tumor and so on. Unlike other cell therapies, the therapeutic effect of MSCs is not only dependent on cell-cell contact, but also the so-called hit-and-run mechanism may exist (165). Cell-to-cell contact occurs by forming gap junctions or tunneling nanotubes with adjacent or nearby cells, which allows for the transfer of small molecules, peptides and organelles (165). Bahr et al. examined autopsy materials from 18 patients who received human leukocyte antigen mismatched mesenchymal stem cells and found that long-term transplanted mesenchymal stem cells appeared to have very low levels at best, due to rejection by the recipient’s immune system, or failure to survive and transplant after intravenous injection (166). They therefore proposed that MSCs appear to regulate their function through a “hit and run” mechanism, rather than through continuous implantation in damaged tissues. While mesenchymal stem cells perform therapeutic functions through a brief “hit and run” mechanism, protecting mesenchymal stem cells from immune detection and prolonging their persistence *in vivo* may improve clinical outcomes and prevent patients from being allergic to donor antigens (167). Therefore, MSCs secrete soluble cytokines, growth factors, hormones and miRNAs in a paracrine manner. Moreover, studies have shown that MSC-derived EVs retain the biological characteristics of parental MSCs and show similar therapeutic effects in some animal models (148).

Up to now, dozens of MSCs therapies have been approved globally. Alofisel is the first allogeneic stem cell therapy approved in the European Union. Adult Crohn disease (CD) patients with refractory, draining, and complex perianal fistulas who received a single intralesion injection of 120 million allogenic AT-MSC (Alofisel) showed marked remission, with a higher remission rate at 52 weeks of follow-up than placebo (60). Despite the success of some MSCs therapies, there are still some factors that may lead to unsatisfactory clinical outcomes, such as MSCs product quality, management, and host factors. Since MSC preparation involves deep cryopreservation until thawing of MSCs at the bedside and infusion into the patient. Studies have shown that cryopreserved MSCs have decreased ability to suppress immunosuppression and inhibit T cell proliferation (168). Different modes of administration can produce different pharmacokinetics (169, 170). Hofmann et al. reported that less than 5% of the injected cells remained at the injection site several hours after local administration of MSCs therapy (171). Therefore, in order to improve the therapeutic effect, the retention and survival of MSC should be further strengthened. To improve local delivery of MSCs, Hu et al. used hypoxic preconditioning of MSCs, which showed increased angiogenesis after transplantation into a myocardial infarction (MI) model compared to normoxic MSCs, and hypoxic preconditioning enhanced the ability of MSCs to repair infarcted myocardium (172). Encapsulation of MSCs by biomaterials can improve its retention and survival ability *in vitro*, but further studies are needed to investigate its effect *in vivo*. Guiding the host to establish a better microenvironment for MSCs therapy can improve its therapeutic efficacy. For example, the water-soluble antioxidant vitamin C can prevent oxidative stress and reduce damage to transplanted cells (168).

MSCs therapy still faces great challenges, and continuing to explore engineering approaches to meet these challenges can improve its clinical indications and enhance its therapeutic efficacy. Enhancing the potency of MSCs through engineering strategies such as small molecule priming, membrane particles engineering, and genetic modification provides a measurable property that can be tested at all stages of preclinical and clinical development, from well-defined potency assays to therapeutic biomarkers in human clinical studies.

## 6 Conclusion

Despite the great advances in medicine over the past few decades, organ transplantation still faces significant obstacles. Alternative strategies for allogeneic organ transplantation are currently being explored. MSCs from autologous and allogeneic sources are used as a cell therapy to reduce the use of immunosuppressive drugs in organ transplant recipients. 3D bioprinting has been applied in tissue engineering and regenerative medicine using 3D printing techniques to print tissues or organs (173). MSCs isolated from various sources have the ability to differentiate into a variety of cells and anti-inflammatory and immunomodulatory properties, which enable them to be used as cell therapy for organ preservation and transplantation, depending on the rapid advances in the fields of tissue engineering and regenerative medicine (174). MSCs can inhibit the activation of cells of the adaptive immune system and innate immune system and convert them into regulatory cells, thereby reducing the host response to the graft. In a corneal transplant model, MSCs infusion protected against allograft rejection by shifting macrophages toward the M2 phenotype (175). Takebe et al. generated liver organoids by co-culturing human pluripotent stem cells, mesenchymal stem cells and human umbilical vein endothelial cells to form 3D liver bud like structures (176). When transplanted into nude mice, the organoids showed functional angiogenesis and drug metabolic activity. Mesenchymal stem cells have a particular impact on aspects of transplantation and regenerative medicine, and MSCs are emerging as a powerful tool in the medical field by improving organ preservation and immune tolerance.

In this review, we summarize the current status of mesenchymal stem cell therapy and the challenges in translational application of clinical therapy. Various biological, biochemical and biophysical factors affect the survival and homing ability of MSCs through the interplay between cells, extracellular matrix and bioactive factors *in vitro* and *in vivo* (177). Therefore, by regulating the above factors to reduce cell damage, improve the survival rate of MSCs, increase the homing ability of MSCs and improve the efficiency of MSCs implantation. For the large-scale clinical production and use of MSCs, it is important to standardize and optimize the cell source and culture price adjustment, formulate more detailed and accurate identification criteria, clarify the biological mechanism, and formulate a standardized plan for MSCs source and combined biomaterials according to different types of diseases, so that MSCs can be clinically transformed as soon as possible.

## Author contributions

RM: Writing – original draft. ZW: Writing – original draft. CY: Writing – original draft. XS: Writing – original draft. RW: Writing – original draft. HZ: Writing – original draft. RY: Writing – original draft. JL: Writing – original draft, Writing – review & editing. YS: Writing – original draft, Writing – review & editing. HS: Writing – original draft, Writing – review & editing.
